# Supplementary material for: Piezoresistive Multi-Walled Carbon Nanotube/Epoxy Strain Sensor with Pattern Design
Source: Materials (Basel). 2019 Nov 29;12(23):3962. doi: 10.3390/ma12233962 (PMC6926730; doi:10.3390/ma12233962)
Supplement: Supplementary file 1 [file materials-12-03962-s001.pdf]

materials

Type of the Paper (Article)

Piezoresistive Multi-Walled Carbon Nanotube/Epoxy Strain Sensor with Pattern Design

Mun-Young Hwang [1,2,†], Dae-Hyun Han[1,2,†] and Lae-Hyong Kang [1,2,3,*]

[1] Department of Mechatronics Engineering, Jeonbuk National University, 567 Baekje-daero, Deokjin-gu, Jeonju-si 54896, Korea; munyoung.h@jbnu.ac.kr (M.–Y.H.); dh.han@jbnu.ac.kr (D.–H.H.)
[2] LANL-JBNU Engineering Institute-Korea, Jeonbuk National University 567 Baekje-daero, Deokjin-gu, Jeonju-si 54896, Korea
[3] Department of Flexible and Printable Electronics, Jeonbuk National University 567 Baekje-daero, Deokjin-gu, Jeonju-si 54896, Korea
* Correspondence: reon.kang@jbnu.ac.kr; Tel.: +82–63–270–3372
† Mun-Young Hwang and Dae-Hyun Han contributed equally to this work.

Received: 31 October 2019; Accepted: 28 November 2019; Published: date

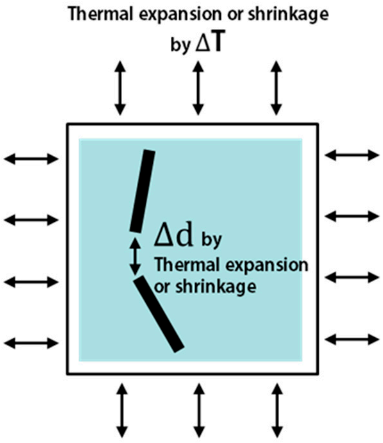

Figure S1. Variation in distance between particles due to thermal expansion or contraction of polymer according to temperature change.

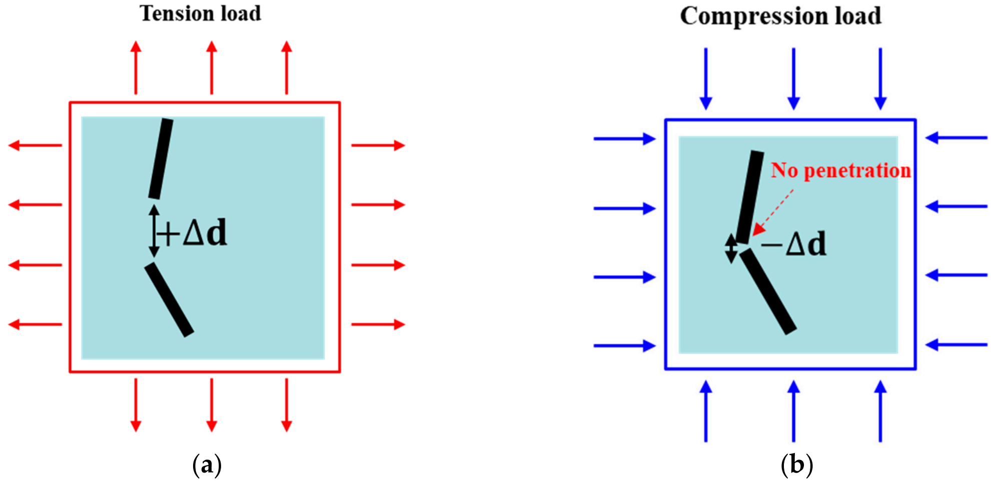

Figure S2. Operating mechanism of composite strain sensor by resistivity (**a**) under tension load and (**b**) compression.

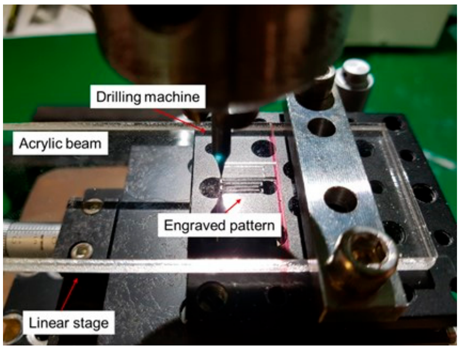

Figure S3. Engraved pattern made by drilling machine and linear stage for making constant pattern.

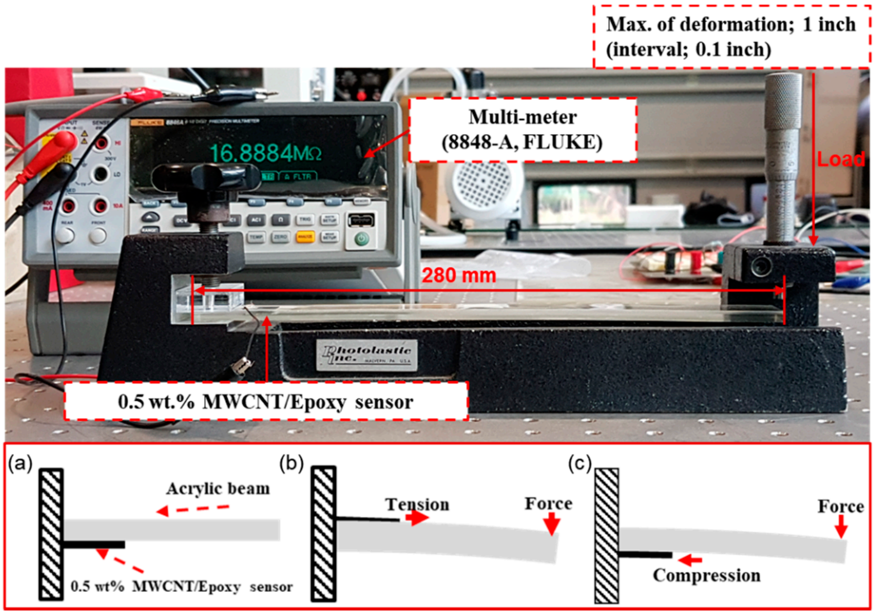

A

Figure S4. Resistance variation of MWCNT/epoxy composite strain sensor when deflection occurs at beam end: (**a**) stead state, (**b**) tension direction, and (**c**) compression direction.

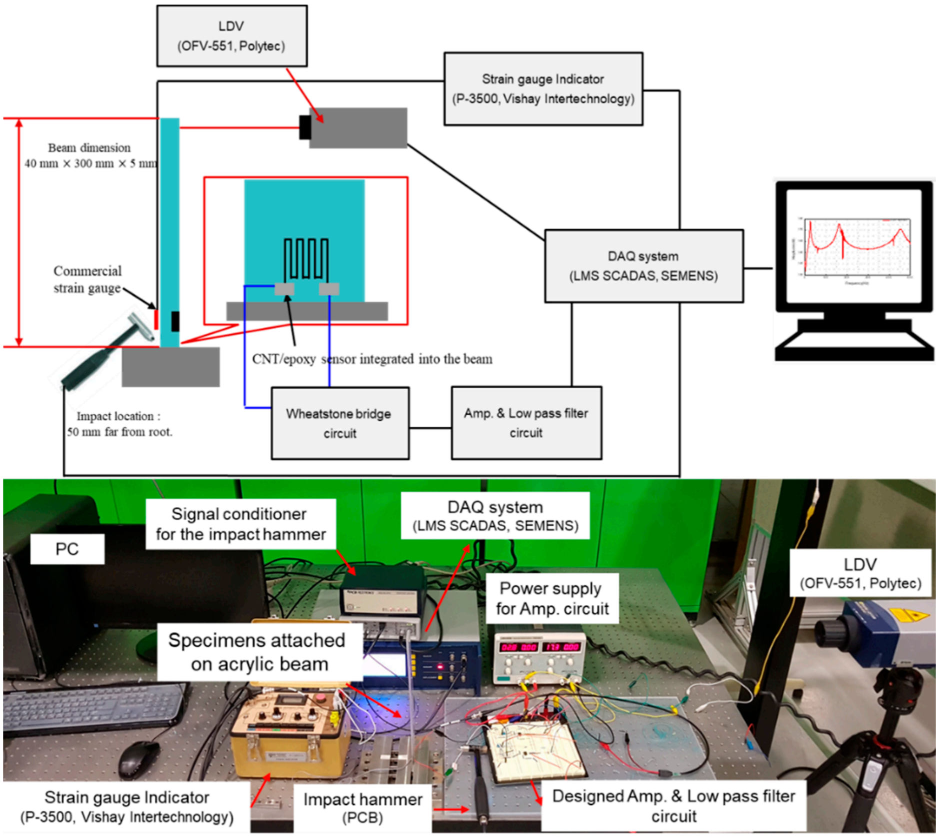

Figure S5. Experimental setup for testing measurement frequency response of sensor under free vibration of acrylic beam.

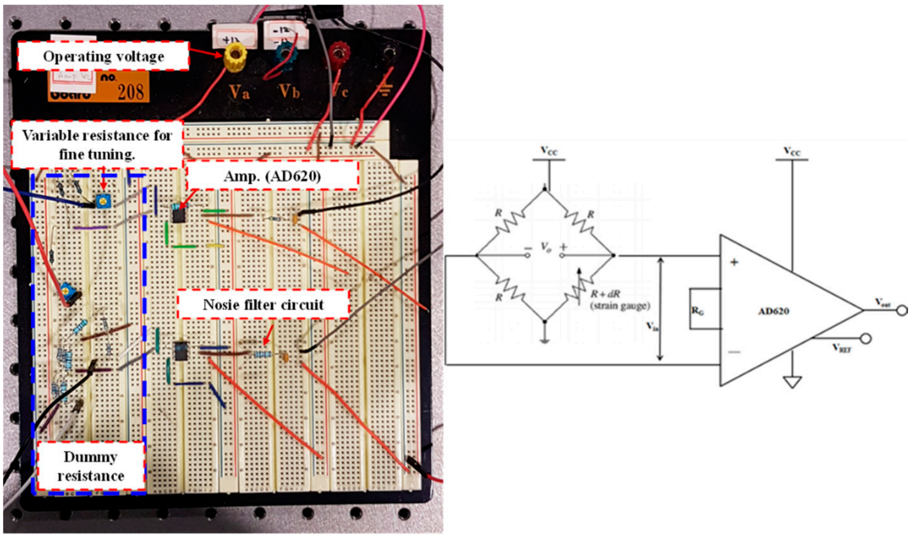

Figure S6. Amplifier circuit by non-inverter amplification for signal processing and Wheatstone bridge circuit for stabilizing output voltage generated by sensor.
